# Supplementary material for: Effectiveness and Safety of Ayurvedic Medicines in Type 2 Diabetes Mellitus Management: A Systematic Review and Meta-Analysis
Source: Front Pharmacol. 2022 Jun 8;13:821810. doi: 10.3389/fphar.2022.821810 (PMC9213670; doi:10.3389/fphar.2022.821810)
Supplement: Supplementary file 1 [file DataSheet1.zip › Supplementary Material/Forest and Funnel Plots/Nigella sativa/HbA1c.pdf]

Nigella sativa - HbA1c

Study	Intervention N	Intervention Mean	Intervention SD	Comparator N	Comparator Mean	Comparator SD	Mean Diff. with 95% CI	Weight (%)
Najmi 2012	40	6.99	0.83	40	7.18	0.70	-0.19 [-0.53, 0.15]	27.25
Hosseini 2013	35	8.52	0.68	35	8.70	0.67	-0.18 [-0.50, 0.14]	28.64
Heshmati 2015	36	7.80	0.80	36	8.60	1.00	-0.80 [-1.22, -0.38]	22.21
Kaatabi 2015	51	7.89	1.29	52	8.27	0.87	-0.38 [-0.80, 0.04]	21.89
**Overall**							-0.36 [-0.64, -0.09]	

Heterogeneity: $\tau^2 = 0.04$, $I^2 = 53.94\%$, $H^2 = 2.17$

Test of $\theta_i = \theta_j$: Q(3) = 6.42, p = 0.09

Test of $\theta = 0$: z = -2.62, p = 0.01

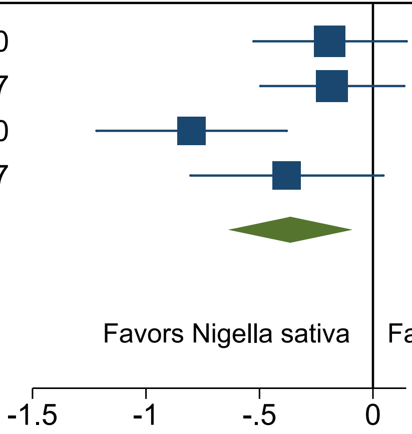

Random-effects REML model
